# Large-Scale Absence of Sharks on Reefs in the Greater-Caribbean: A Footprint of Human Pressures

**DOI:** 10.1371/journal.pone.0011968

**Published:** 2010-08-05

**Authors:** Christine A. Ward-Paige, Camilo Mora, Heike K. Lotze, Christy Pattengill-Semmens, Loren McClenachan, Ery Arias-Castro, Ransom A. Myers

**Affiliations:** 1 Department of Biology, Dalhousie University, Halifax, Nova Scotia, Canada; 2 Reef Environmental Education Foundation, Key Largo, Florida, United States of America; 3 Department of Biological Science, Florida State University, Tallahassee, Florida, United States of America; 4 Department of Mathematics, University of California San Diego, San Diego, California, United States of America; National Oceanic and Atmospheric Administration/National Marine Fisheries Service/Southwest Fisheries Science Center, United States of America

## Abstract

**Background:**

In recent decades, large pelagic and coastal shark populations have declined dramatically with increased fishing; however, the status of sharks in other systems such as coral reefs remains largely unassessed despite a long history of exploitation. Here we explore the contemporary distribution and sighting frequency of sharks on reefs in the greater-Caribbean and assess the possible role of human pressures on observed patterns.

**Methodology/Principal Findings:**

We analyzed 76,340 underwater surveys carried out by trained volunteer divers between 1993 and 2008. Surveys were grouped within one km^2^ cells, which allowed us to determine the contemporary geographical distribution and sighting frequency of sharks. Sighting frequency was calculated as the ratio of surveys with sharks to the total number of surveys in each cell. We compared sighting frequency to the number of people in the cell vicinity and used population viability analyses to assess the effects of exploitation on population trends. Sharks, with the exception of nurse sharks occurred mainly in areas with very low human population or strong fishing regulations and marine conservation. Population viability analysis suggests that exploitation alone could explain the large-scale absence; however, this pattern is likely to be exacerbated by additional anthropogenic stressors, such as pollution and habitat degradation, that also correlate with human population.

**Conclusions/Significance:**

Human pressures in coastal zones have lead to the broad-scale absence of sharks on reefs in the greater-Caribbean. Preventing further loss of sharks requires urgent management measures to curb fishing mortality and to mitigate other anthropogenic stressors to protect sites where sharks still exist. The fact that sharks still occur in some densely populated areas where strong fishing regulations are in place indicates the possibility of success and encourages the implementation of conservation measures.

## Introduction

**Figure 1:**
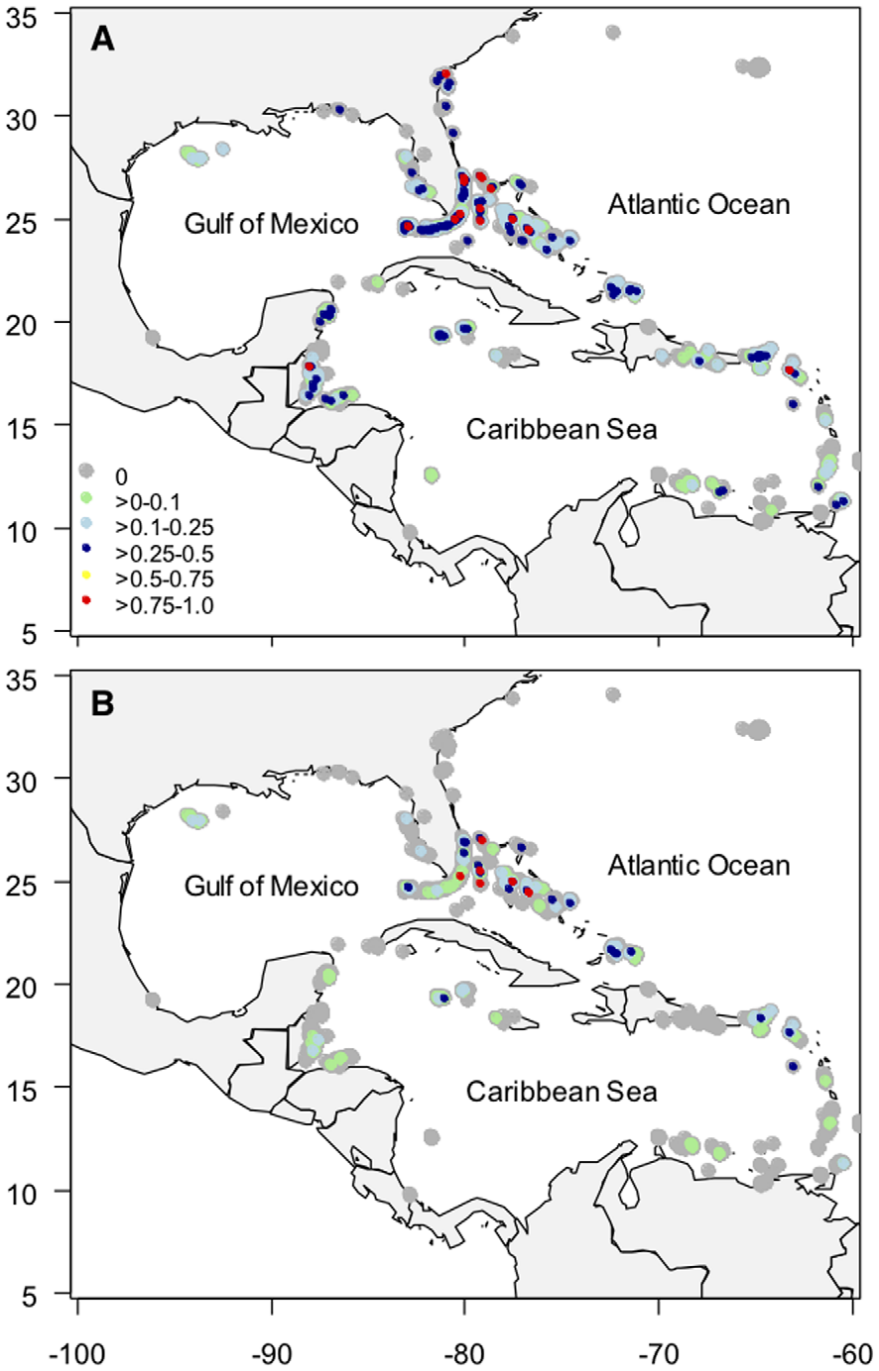
Distribution and sighting frequency of sharks on reefs in the greater-Caribbean. Shown are sampled 1 km^2^ cells for A) all species combined, and B) all species excluding nurse sharks (*Ginglymostoma cirratum*). Note that cells were enlarged for the patterns of distribution to be seen at this scale.

Strong declines in the abundance of many large pelagic sharks have been described worldwide and repeatedly linked to industrial fishing [1]–[5]. The extent of these declines and some of their ecosystem consequences have been described with the use of long-term catch datasets, mostly in pelagic systems [6]. Unfortunately, the status of shark populations in other ecosystems, such as coral reefs, remains poorly known because both modern and historical data are very limited [7]. This uncertainty, in combination with the high vulnerability of sharks to fishing [3], [8] has motivated the use of alternative sources of data to shed light on temporal and spatial trends in shark populations. These sources of data include historical fisheries and market records of sharks in the Mediterranean [9], trophy photographs of fishing tournaments in Florida [10], archaeological and historical records on coral reef ecosystem changes worldwide [11], and ecological surveys of fish communities across spatial gradients of exploitation [12]–[14], among others. Although these analyses have been opportunistic and restricted to few regions they have been valuable in describing changes in populations. Here, we explore another source of data based on observations made by trained scuba divers to examine patterns of distribution and sighting frequency of sharks on reefs in the greater-Caribbean, which includes sites in the western central Atlantic from northern Florida to northern Brazil, the Gulf of Mexico and the Caribbean Sea (Fig. 1).

Data collected by trained recreational divers can be a reliable and valuable source of data for describing large-scale patterns on the status of sharks. Trained scuba divers have been shown to collect data that is comparable to scientific divers across a range of biological metrics [15]–[17]. Although trained divers are instructed in the identification of a broad-range of fish species, the identification of sharks may be poor since many species are morphologically similar; however, data can still be reliable when looking at patterns of sharks as a group given the ease of differentiating sharks from other fish. Recreational divers travel to many sites providing data from a range of locations and allowing an overview of trends over large-spatial scales while the robustness of the emerging patterns can be supported by the sheer number of observations (i.e. number of divers and dives). Because recreational divers survey a wide range of habitats, depths, and times of the year, they also maximize the sighting probability of less common taxa such as sharks. This non-extractive sampling technique allows data to be obtained for animals that are at risk and from areas where fishing is prohibited. Sites preferred by recreational divers generally have more abundant large fish [18] and are likely inversely related to commercial fishing pressure, therefore providing a conservative look at the state of sharks.

Sharks on reefs can be particularly vulnerable to the growth and spatial expansion of human populations. As a group, sharks are susceptible to even mild levels of fishing mortality given their late age of maturity, slow growth, and slow reproductive rate [3], [8]. In the greater-Caribbean, there has been a long and ongoing history of exploitation of sharks (see Table 1), which accelerated during the 20^th^ century [11], [19]. Reef sharks, including tiger (*Galeocerdo cuvier*), lemon (*Negaprion brevirostris*), sandtiger (*Carcharias taurus*) and silky (*Carcharhinus falciformis*) began to be targeted intensively in the early 20^th^ century for their liver, skins, meat and fins [19] and commercial catches increased after 1950 [2], [20], [21]. Landings of sharks more than tripled in the Gulf of Mexico between 1980 and 1989 and Caribbean elasmobranch landings peaked at more than 9 million metric tons in 1990 [22]. Excessive harvesting of juveniles in recent years likely has exacerbated the effects of decades of fishing [20], [23] – a trend which coincides with the increased demand for shark fins in Asian markets [24]. Beyond direct exploitation, the presence of human settlements can lead to habitat degradation and destruction [25], which can reduce the area that is suitable for sharks and their survival rates [26]. Similarly, overfishing of reef fish, which is related to human population density [27], may reduce the prey population available to sharks. As well, other anthropogenic stressors often occur simultaneously and likely act synergistically to exacerbate the loss of sharks; however, this has not been adequately quantified and trends in abundance and distribution of sharks on reefs are urgently needed to substantiate the establishment of conservation strategies.

**Table 1:** Some historical narratives on the abundance of sharks in the greater-Caribbean.

Comment	Area	Period
The earliest written observations of sharks appeared during Columbus' voyages, when a large group of sharks surrounded the explorers' ships off the east-coast of Panama. The sailors were frightened by the number and ferocity of the sharks and “made carnage among them with a chain hook until [sailors] could kill no more” [53].	Panama	1500's
The naturalist Hans Sloane wrote of numerous encounters with sharks off the coast of Jamaica, and noted that it was “ordinary to have sharks come about the ships” [54]; this statement certainly could not be made today, since Jamaica has one of the most depauperate fish populations in the Caribbean [55].	Jamaica	1680's
It was common for sharks to “swarm about the wharves, feeding on refuse fishes” in the Florida Keys [56].	Florida Keys, USA	1880's
Sharks were described as “plentiful” [57] and “one of the most common types of fish” throughout the Leeward Antilles [58].	Antillles	1880's
In the Florida Keys, daily catches of 50–100 sharks, consisting mostly of leopard (tiger), dusky, hammerhead, sand (sandtiger), and nurse sharks, were made with nets in just 15 ft (4.57 m) of water [47], [59] – well within the depth range of divers.	Florida Keys, USA	1920's
Baughman and Springer [51] described how sharks were so abundant that they were “expected anywhere at anytime” in the west-Indian Caribbean.	West-Indian	1950's
Data on recreational fishing for the Atlantic and Gulf of Mexico indicate yearly catches of up to 1,5 million coastal and pelagic sharks for the period 1974–75 alone [60].	Atlantic & Gulf of Mexico	1970's

In the greater-Caribbean, poor knowledge of the status of sharks on reefs is worrisome given the long history of reef exploitation (Table 1) [28], the extensive distribution of human settlements in the region [29] and the need to substantiate the establishment of conservation strategies. Here, we used a broad and comprehensive collection of underwater surveys conducted by trained divers in the greater-Caribbean to explore contemporary patterns in shark distribution and sighting frequency. We then assessed the role of anthropogenic stressors by comparing the sighting frequency of contemporary sharks to human population density. Finally, we used population viability analysis to assess the specific effect of fishing mortality on the distribution and sighting frequency of sharks.

## Methods

### Determining the distribution and sighting frequency of sharks

We used underwater visual censuses to describe the contemporary distribution and sighting frequency of sharks on reefs in the greater-Caribbean. Surveys were conducted by trained volunteer divers between 1993 and 2008 for the Reef Environmental Education Foundation (REEF, www.reef.org). Using the Roving Diver Technique (RDT) [30] divers survey a wide variety of habitats within a particular site and record all fish, including sharks, that are observed throughout the water column during their regular dive activities. This database contains ∼100,000 surveys broadly distributed throughout the greater-Caribbean.

For the purpose of this study habitats were limited to reefs (high and low profile), slopes (dropoff, wall, ledge) and flats (grass, sand, rubble) – termed ‘reef’ from here on (for more habitat information see www.reef.org). Open water and artificial habitats were excluded. Sites with geo-referenced locations (latitude and longitude) were allocated into 1 km^2^ cells. In the REEF database abundance is recorded as binned values (e.g. 1 = 1, 2 = 2–10, 3 = 11–100) and original counts are unknown. Therefore, to eliminate any error associated with the number counted we limited our analyses to presence or absence of sharks on each dive. For each cell with more than 5 dives, we quantified the sighting frequency as the number of dives where sharks were reported divided by the total number of dives within that cell. To determine if there was a consistent bias in our results due to preference or indifference for diving with sharks, we used a regression model to compare the sighting frequency of sharks with survey effort (i.e. number of dives).

We limited our analyses to species that are described as reef-dwelling or reef-associated in Compagno et al. [31] and included bonnethead (*Sphyrna tiburo* Linnaeus), blacknose (*Carcharhinus acronotus* Poey), Atlantic sharpnose (*Rhizoprionodon terraenovae* Richardson), sandtiger (*Carcharias taurus* Rafinesque), blacktip (*Carcharhinus limbatus* Müller and Henle), tiger (*Galeocerdo cuvier*), spinner (*Carcharhinus brevipinna* Péron and Lesueur), silky (*Carcharhinus falciformis* Müller and Henle), lemon (*Negaprion brevirostris* Poey), bull (*Carcharhinus leucas* Müller and Henle), sandbar (*Carcharhinus plumbeus* Nardo), nurse (*Ginglymostoma cirratum* Bonnaterre), whale (*Rhincodon typus* Smith), Caribbean reef (*Carcharhinus perezii* Poey), scalloped hammerhead (*Sphyrna lewini* Griffith and Smith), great hammerhead (*Sphyrna mokarran* Rüppell), and smooth hammerhead (*Sphyrna zygaena* Linnaeus). Compagno [31] does not refer to the dusky shark (*Carcharhinus obscurus*) as reef associated, and was therefore excluded from our analyses. If this species had been misidentified and was one of the species that we analyzed, its inclusion would have had a negligible effect on our results as all data for this species consisted of only 16 observations at one site in the Texas Flower Garden Banks, in the northern Gulf of Mexico. Many shark species overlap in distribution, habitat use, and have similar morphologies, which can make identification difficult during field observations. We therefore used a cautionary approach, assuming all species could be misidentified, and combined the records for all these species. As such, our patterns should be interpreted for all sharks on reefs in general. However, we performed an additional analysis excluding nurse sharks, which are stationary, relatively common and reasonably easy to identify, and is the species with the least commercial value today [32]. We performed this additional analysis because the differences introduced by this single, relatively unexploited species may reveal the effects of targeted fishing on the loss of sharks due to their commercial value.

### Comparing patterns of shark sightings to human population density

The growth and spatial expansion of human populations have been accompanied by changes in land use, pollution and exploitation of natural resources [25]. As such human population density can serve as a proxy for multiple anthropogenic stressors and was used here as a metric to assess the potential effect of human pressures on sharks. Specifically, we compared the sighting frequency of i) all sharks, ii) all sharks excluding nurse sharks and iii) only nurse sharks to the number of humans nearby. Data on human population were available at a resolution of 2.5′ (∼5 km^2^ cells) for the year 2000 and were obtained from http://sedac.ciesin.columbia.edu/gpw/global.jsp. For each of the one km^2^ cells where the dive surveys were grouped, we added a 10 km buffer and obtained the maximum number of people within that buffer. The 10 km buffer was chosen as an easy to travel distance for fishers and a distance where reef habitats may be strongly influenced by land-based human disturbances, such as coastal development and pollution. Correlations between human density and sighting frequency were fitted using inverse power models.

### Population viability analysis and the effect of fishing mortality

To explore the possible effects of exploitation on sharks, we used population viability analysis to assess whether fishing pressure alone could explain the observed patterns of sharks. Such demographic analyses quantify the resilience of species to different levels of fishing mortality given their life history attributes. Life history attributes were obtained from different sources for each species analyzed in the sightings database (see Table 2) and included age at maturity (α), longevity (*w*), fecundity (*b*, i.e. female pups per female per year, which was calculated from data on number of pups, gestation period and reproductive frequency), and natural mortality (*M*). Natural mortality (*M*) was estimated from longevity using Hoenig's [33] formula:
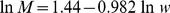
Survival to age at maturity (*l*_α_) was calculated from a variant of the Euler-Lotka equation:
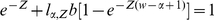
where *l_α,Z_* is survival to age at maturity when total mortality is equal to *Z*. Total mortality (*Z*) is set at twice the natural mortality (this condition is applied to minimize the effects of density dependence) and population growth is stable (*r* = 0) [34]. The intrinsic rate of population increase (*r*) was calculated as the rebound potential [34] or the growth rate of a population in the near absence of density-dependent controls. *r* was calculated as the value that satisfies the following variant of the Euler-Lotka equation:
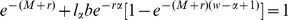
Changes in population size (*N*) due to exploitation were calculated in relative terms for each species using density-dependent and density-independent models of population growth:
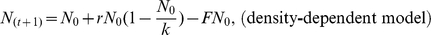


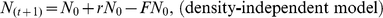
where *F* is fishing mortality or the proportion of the population that is removed by fishing. For one of our studied species (Atlantic sharpnose shark), *F* has been estimated at 0.46 in the greater-Caribbean [35]. Therefore, to assess population viability of the different species under a range of fishing mortalities, *F* was set from 0 to 0.5, increasing at an interval of 0.1. Given that models were run in relative terms, N_0_ and carrying capacity (*k*) were set to 1. All parameters were calculated and represented graphically in a macro in Microsoft Excel which is available upon request. As a precautionary note, population viability analyses exclude some of the complexities of real ecosystems and therefore tend to predict high risk of decline; yet, they “can be useful for screening-level assessments, which should in general be precautionary” [36]. For reef sharks, population viability analysis may provide a good approximation of population trends because many are exploited throughout their entire life cycle [20], [37], [38].

**Table 2:** Life history attributes of analyzed shark species and calculated values for natural mortality, survival to age at maturity and intrinsic rebound potential (body size values from Compagno et al.[31], other data summarized in Frisk et al. [61] except scalloped hammerhead [38], nurse [32], [38] and whale shark [38], [62]).

		Litter Size									
Common Name	Latin Name	Min	Max	Fecundity period (Years)	Average female pups per female per year	Age at maturity (Years)	Longevity (Years)	Survival until maturity age	Natural mortality	Body Size (cm)	Intrinsic rebound potential
Bonnethead	*Sphyrna tiburo*	3	15	1	4.5	2.5	7	0.16	0.62	150	0.117
Blacknose	*Carcharhinus acronotus*	3	6	1	2.3	3	4.5	0.38	0.96	200	0.070
Atlantic sharpnose	*Rhizoprionodon terraenovae*	1	12	1	3.3	4	10	0.18	0.44	110	0.084
Sandtiger	*Carcharias taurus*	2	2	2	0.5	6	17	0.82	0.26	430	0.062
Blacktip	*Carcharhinus limbatus*	1	11	2	1.5	7	18	0.26	0.25	225	0.054
Tiger	*Galeocerdo cuvier*	10	82	2	11.5	9	28	0.02	0.16	750	0.043
Spinner	*Carcharhinus brevipinna*	6	10	2	2	7.5	12	0.27	0.37	278	0.045
Silky	*Carcharhinus falciformis*	10	12	1	5.5	9	25	0.06	0.18	330	0.043
Lemon	*Negaprion brevirostris*	4	17	2	2.6	12.7	25	0.12	0.18	340	0.032
Bull	*Carcharhinus leucas*	6	12	2	2.3	15	27	0.12	0.17	340	0.027
Scalloped Hammerhead	*Sphyrna lewini*	12	41	1	13.3	15	30.5	0.02	0.15	420	0.028
Sandbar	*Carcharhinus plumbeus*	5	12	2	2.1	15	30	0.12	0.15	240	0.028
Nurse	*Ginglymostoma cirratum*	21	50	2	8.9	15	25	0.03	0.18	430	0.026
Whale	*Rhincodon typus*	16	300	2.5	31.6	21	100	0.00	0.04	2100	0.017

Note: We did not include Caribbean reef (*C. perezii*), great hammerhead (*S. mokarran*) or smooth hammerhead (*S. zygaena*) because of missing life history data.

## Results

### Distribution and sighting frequency of sharks on reefs in the greater-Caribbean

In total we analyzed 76,340 dives across 1,382 one km^2^ cells, with an average of 55 (S.E. ±3.3) dives per cell. Sharks were observed in 762 cells (i.e. 55% of all cells with more than 5 dives; Fig. 1A). Of these, 441 (32%) cells contained only nurse sharks, 227 (16%) contained a mixture of nurse and other sharks and 94 (7%) contained sharks other than nurse sharks. Across all cells, the average sighting frequency of sharks (i.e. the fraction of dives in which sharks were sighted) was 10% (S.E. ±0.004) for all sharks and 3% (S.E. ±0.003) for all sharks excluding nurse sharks.

The pattern of shark distribution in the greater-Caribbean was clearly affected by the inclusion of nurse sharks. When nurse sharks are considered, sharks were observed on reefs throughout most of the greater-Caribbean at some time during the study period (Fig. 1A). The greatest concentration of cells with high sighting frequency occurred in the Bahamas, southeastern US and Belize. With the exclusion of nurse sharks, however, the number and range of cells where sharks occurred was much smaller (Fig. 1B). Notably, sharks other than nurse sharks were largely absent in cells around Cuba, Jamaica, Dominican Republic, Puerto Rico, throughout most of the Antilles and central and South America. The greatest concentration of cells with sharks, other than nurse sharks, occurred in the Bahamas.

**Figure 2:**
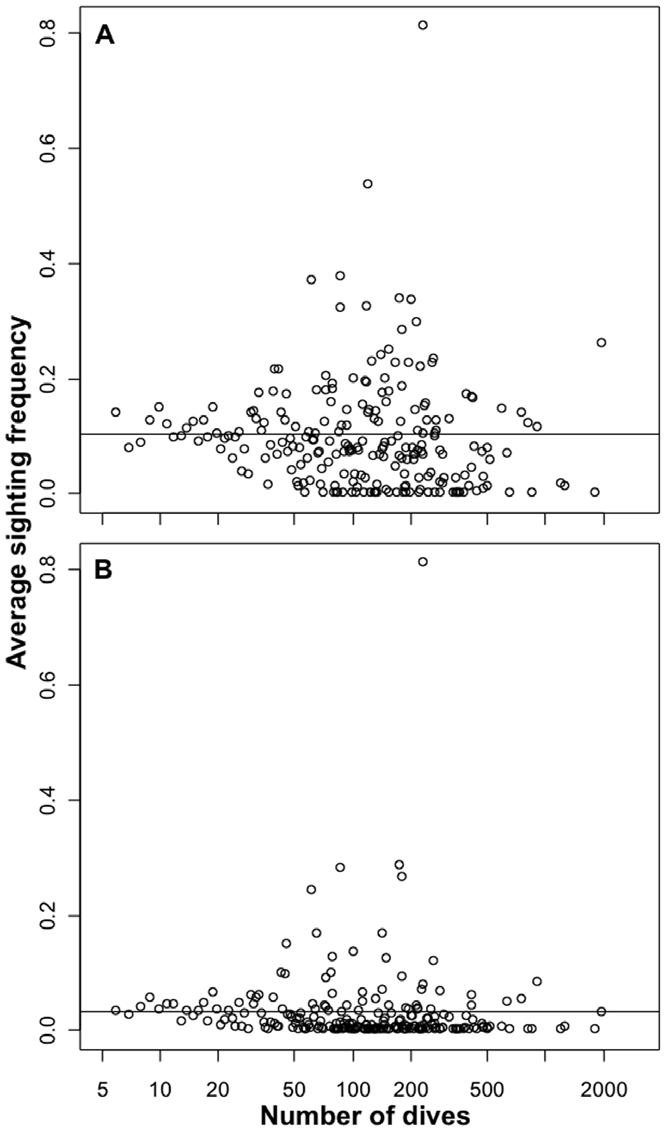
Effect of sampling effort on sighting frequency. Relationship between the number of dives per 1 km^2^ cell and the sighting frequency of A) all sharks (r^2^ = 0.003, p = 0.4), and B) all sharks excluding nurse sharks (r^2^ = 0.0005, p = 0.4). Solid lines are linear models showing there is no change in sighting frequency with effort.

Although there was high variability in sighting frequency at intermediate levels of effort (i.e. 50–500 dives), there were no significant trends of sighting frequency with survey effort for all sharks (slope = −0.002, S.E. = 0.003, p = 0.28) or for all sharks excluding nurse sharks (slope = −0.002, S.E. = 0.002, p = 0.41) (Fig. 2). This suggests that variation in sampling effort (dives per cell) did not affect sighting frequency and should therefore not affect our results. In other words, sighting frequencies were on average similarly low using 10 or 500 dives in a cell (Fig. 2).

### Effect of human population density on the sighting frequency of sharks

**Figure 3:**
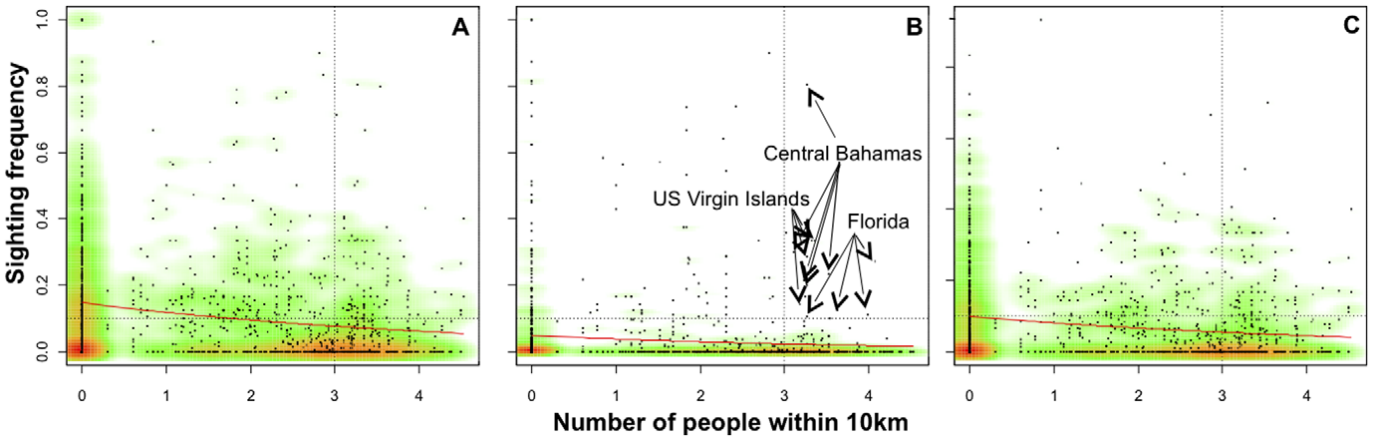
Relationship between sighting frequency of sharks in surveyed 1 km^2^ cells and the log of the number of people within a 10 km radius of each cell for A) all shark species combined, B) all sharks excluding nurse sharks (*Ginglymostoma cirratum*), and C) nurse sharks only. Data on the number of people was obtained from the Socioeconomic Data and Applications Center at http://sedac.ciesin.columbia.edu/gpw/global.jsp. Trends (solid red lines) were fitted using inverse power models to define the relationship between sighting frequency of sharks (y) and log of human density (x). The models were A) y = 1/[6.8*(x+1)^0.09^], p<0.0001; B) y = 1/[20.6*(x+1)^0.11^], p = 0.0002; C) y = 1/[10.2*(x+1)^0.09^], p<0.0001. The graded colours on the figure are used to identify the density of cells on the plots where red = high, green = intermediate, white = low. Dashed lines show human population density = 1000 and sighting frequency = 0.10.

Comparison of the sighting frequency of sharks and human population density showed that, with the exception of nurse sharks and a small number of cells, sharks are absent in the majority of cells and that contemporary sharks occur mostly where human population density is low (Fig. 3). The few cells with a high sighting frequency of sharks (>10%) and large human population (>1000 people) occurred in Florida (4 cells), central Bahamas (4 cells), and the U.S. Virgin Islands (3 cells), which are areas that have strong fishing regulations such as prohibition of shark finning, extensive marine protected areas, and in the case of the Bahamas, prohibition of gillnet and long-line fishing. Based on AIC model selection, we found that inverse power models fit the relationship between human population density and shark sighting frequency better than inverse exponential models. Consequently, we report the results of the former only. For all the patterns analyzed, power models were highly significant (see Fig. 3A–C) and indicate that the sighting frequency of sharks was high only in areas where human population was very low (Fig. 3A–C).

### Population viability analysis and the effect of fishing mortality

**Figure 4:**
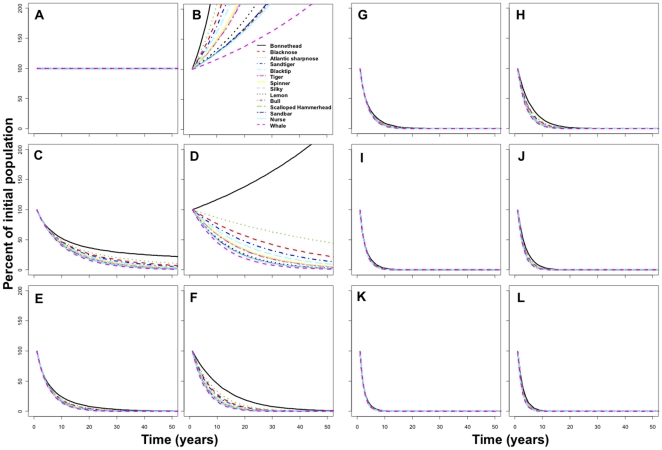
Absolute changes in population abundance of 14 shark species (see color code) across a range of fishing mortalities. Density dependent (left column) and density independent (right column) scenarios are shown with fishing mortality values (*F*): *F* = 0 (A, B), *F* = 0.1 (C, D), *F* = 0.2 (E, F), *F* = 0.3 (G, H), *F* = 0.4 (I, J), *F* = 0.5 (K, L).

As expected, under scenarios of zero fishing mortality (*F* = 0) populations under density dependence remained stable at carrying capacity (Fig. 4A) and increased under density independence (Fig. 4B). Under fishing mortalities of *F* = 0.1, where 10% of the population is removed per year due to fishing, all species declined to between 1 and 14% of their initial population size within 50 years under density dependent conditions (Fig. 4C). With the exception of bonnethead sharks, all species showed declining trends with fishing mortalities as low as *F* = 0.1 under density independent conditions (Fig. 4D). For the remaining scenarios of fishing mortality and density dependence all species declined by 99% within 28 years. As fishing mortality increased (Fig. 4E–L), the time to reach 1% of the initial population was reduced markedly. Under the conservative scenario of density independence, fishing mortalities of *F* = 0.2 and *F* = 0.5 reduced the populations of all species to less than 1% of their original population sizes in less than 39 and 10 years, respectively (Fig. 4F–L)

## Discussion

Using an extensive database of fish surveys conducted by trained recreational divers we show that contemporary sharks, other than nurse sharks, are largely absent on reefs in the greater-Caribbean. Comparison with data on human population density suggests that such disappearance may have been related to anthropogenic pressures. Our study revealed that sharks on reefs in the greater-Caribbean occurred mostly in areas with very low human population density or in a few places where strong fishing regulations or conservation measures are in place. Population viability analysis indicates that even low levels of fishing mortality can cause shark populations to decline to a small fraction of their initial abundance within a few decades, and suggests that fishing alone could explain the absence of sharks. These patterns are similar to those observed for other coastal and reef shark populations [12], [14], [39] and may be indicative of a broader trend for regions that have a long history of exploitation.

The link between human population density and the absence of sharks on reefs is likely due to anthropogenic stressors that directly and indirectly affect their populations. Of the species analyzed, the IUCN [38] listed 2 as endangered, 4 as vulnerable, 8 as near-threatened, 2 as least concern and 1 as data deficient (nurse shark) at the global scale. For these species, fishing was identified as the main threat [38], which is corroborated by studies that have demonstrated the extent of overfishing of large predators in the greater-Caribbean [10], [11], [20], [21], [40], [41]. Considering the timescale of exploitation (Table 1) and the results of our population viability analysis which show that even low levels of fishing pressure cause shark populations to be reduced to a small fraction of their initial population within a few decades, it is likely that fishing pressure alone, whether targeted or incidental, could explain the observed large-scale absence of sharks. However, the high vulnerability of sharks to fishing pressure is likely exacerbated by cumulative anthropogenic factors that could reduce shark populations on reefs, such as habitat destruction or degradation [26], [42], [43], climate change [7], [38], [44], [45], and pollution [38].

High vulnerability of sharks to fishing pressure is likely exacerbated by the long history of exploitation and the cumulative human impacts in coastal waters. 2200 years ago, virtually all islands in the Caribbean were already colonized and recreational and artisanal fishing activities on reefs and nearshore habitats have increased and expanded dramatically since then [28], including a demand for different shark products (e.g. meat, oil, skin, fins) that extends to present times [20], [46], [47]. Additional evidence for the role of fishing is our results on nurse sharks. This species has a low rebound potential (Table 2), suggesting that it would be very vulnerable to even mild levels of fishing. However, it is the most frequently sighted shark on reefs today and although there are some differences in the diet, behaviour and habitat use of nurse shark and other sharks, compared to the rest of our analyzed species it is the one that has had the least value for its meat or fins [19], [32] and is often discarded with low post-capture mortality [38]. This suggests that nurse sharks may be the least affected by fishing in our study, which would explain the presence of this species as well as highlighting the likely role of fishing on the absence of targeted sharks on reefs. Nevertheless, even nurse sharks show decreasing sighting frequency with increasing human density suggesting that other human impacts also play a role. Finally, the presence of sharks near populated areas, such as in Florida, the central Bahamas and U.S. Virgin Islands, where strong fishing regulations and large and long-established marine protected areas exist, stresses the importance of fishing as the likely main driver of the observed large-scale absence of targeted sharks as well as the possible success of management and conservation in protecting sharks.

One possible caveat to our analysis regards the quality of the data. It is possible that divers avoid sites with sharks, miss sharks while diving, or that sharks avoid divers. Although we found no bias in the number of dives for or against sites with sharks (Fig 2), if there was a spatial sampling bias it should be towards sites with sharks and other abundant large fish because of their appeal to recreational divers [18], [32], [48]. As well, most sharks are very conspicuous, which makes it unlikely that divers would miss them on a typical dive, especially when using the Roving Diver Technique because they are meant to search the entire water column for as many species as possible. Finally, scientific diver surveys have been previously used to characterize shark populations in other reef areas of the world where sharks are abundant such as in the central Pacific Ocean [13], [14], [49], Andaman Sea [50], and Great Barrier Reef [12], supporting the reliability of diver data for assessing spatial trends in shark sightings. Moreover, all of these studies found a strong negative relationship of sharks across a spatial gradient of human population density or exploitation [12]–[14], corroborating our results.

**Figure 5:**
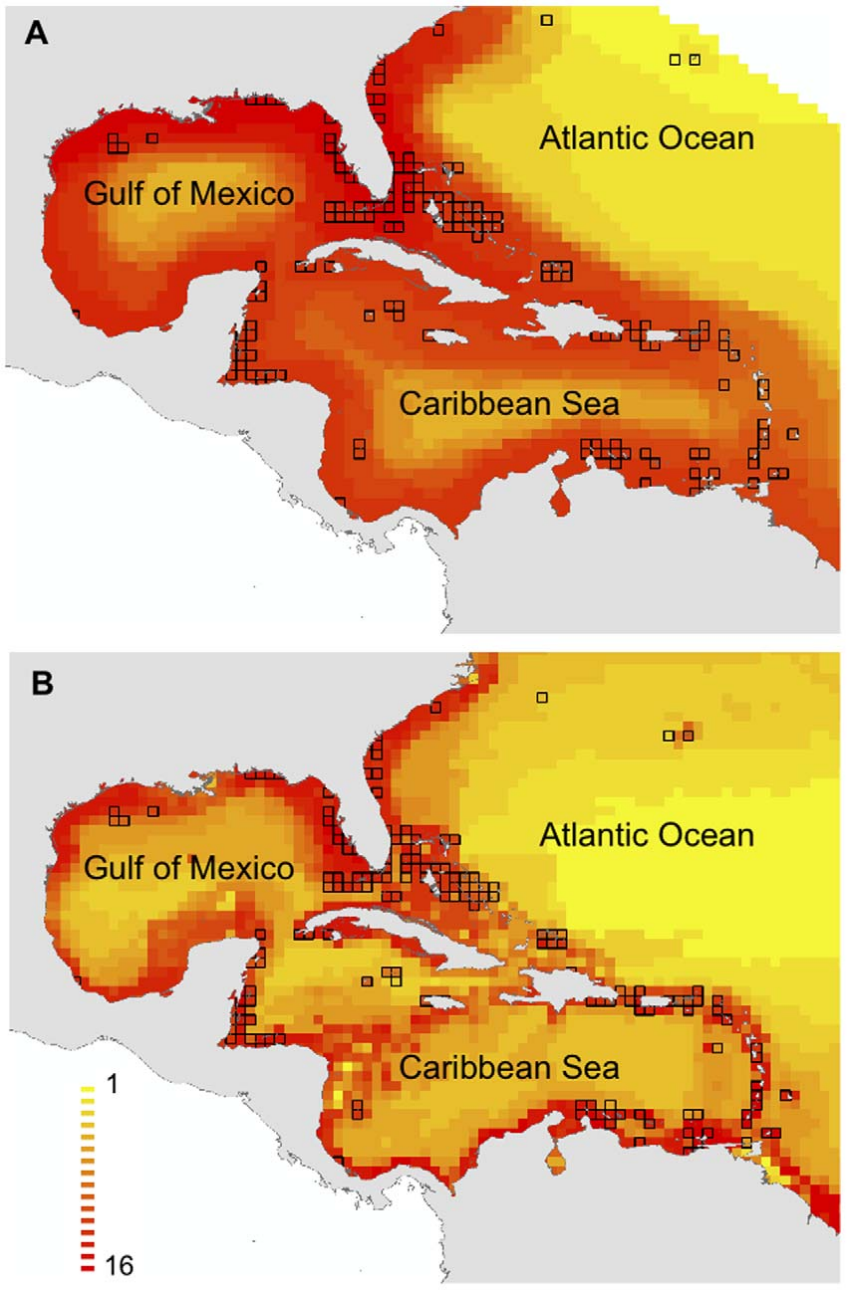
Patterns of species richness for the analyzed shark species. Here we show the number of shark species (see color code from 1 to 16) whose (A) geographical ranges and (B) suitable habitats overlap in half-degree cells across the greater-Caribbean. Geographical ranges were obtained from Compagno et al. [31] and suitable habitats for each species from AquaMaps [52]. Suitable habitats are based on the envelope of the environmental conditions where each species has been reported; the variables considered include depth, temperature, salinity, primary productivity, and distance to coastal areas. Cells containing diver survey data from our study are outlined with black borders.

A second possible caveat of the interpretation of our results is that sharks never existed in these areas or that they occurred at such low densities that they were missed by divers. However, geographical ranges, based on expert opinions and fisheries data [31], indicate that the analyzed shark species should occur throughout the study area (Fig. 5A). Furthermore, the entire study area encompasses habitats of suitable environmental conditions for the presence of the analyzed shark species (see further details in Fig. 5B). We also found numerous records pointing out the generally high abundance of sharks in the greater-Caribbean in the past (Table 1). Although these narratives cannot be directly linked to our study sites, those records that we presume occurred within the depth range of divers and that did not use an attractant (i.e. bait) indicate that sharks, including our studied species, were markedly more abundant than what they appear to be today. Interestingly, Baughman and Springer [51] stated that sharks were “**expected anywhere at anytime**” in the west-Indian Caribbean; in contrast, our analysis of contemporary dive surveys indicate that with the exception of nurse sharks, sharks are **expected anytime almost nowhere**.

Overall, our results indicate that human stressors in coastal areas, potentially dominated by exploitation, have likely led to the absence of shark populations on many reefs in the greater-Caribbean. Contemporary sharks mostly occur in areas with low human populations or where strong fishing regulations or enforced marine reserves occur. Yet historical records, range maps and habitat suitability models suggest that sharks used to, and still could, occur on reefs throughout the greater-Caribbean. Preventing the complete loss of sharks on reefs in the greater-Caribbean requires urgent management measures to mitigate human pressures in coastal waters, especially directed and incidental exploitation from commercial, artisanal and recreational fishing, and protect sites from pollution and habitat destruction where sharks still exist. The fact that sharks still occur in densely populated areas where strong fishing regulations are in place indicates the possibility of success and may encourage the implementation of conservation measures that would restore sharks together with their ecological and functional roles on reefs.
